# Clinical Relevance of the Systematic Analysis of Copy Number Variants in the Genetic Study of Cardiomyopathies

**DOI:** 10.3390/genes15060774

**Published:** 2024-06-13

**Authors:** David de Uña-Iglesias, Juan Pablo Ochoa, Lorenzo Monserrat, Roberto Barriales-Villa

**Affiliations:** 1 Universidad de A Coruña, 15071 A Coruña, Spain; rbarrialesv@gmail.com; 2 Health in Code, 46024 Valencia, Spain; juanpablo.ochoa@healthincode.com; 3 Instituto de Investigación Biomédica de A Coruña (INIBIC), 15006 A Coruña, Spain; 4 Medical Department Dilemma Solutions, 15008 A Coruña, Spain; lorenzo.monserrat@naeviamedical.com; 5 Complexo Hospitalario de A Coruña, Servizo de Saúde (SERGAS), 15006 A Coruña, Spain; 6 Centro de Investigación Biomédica en Red (CIBERCV), 28029 Madrid, Spain

**Keywords:** cardiomyopathies, copy number variants (CNVs), NGS, hypertrophic cardiomyopathy, HCM, dilated cardiomyopathy, DCM, non-compaction cardiomyopathy, LVNC, arrhythmogenic right ventricular dysplasia, ARVC, restrictive cardiomyopathy, RCM

## Abstract

Cardiomyopathies (CMs), one of the main causes of sudden death among the young population, are a heterogeneous group of myocardial diseases, usually with a genetic cause. Next-Generation Sequencing (NGS) has expanded the genes studied for CMs; however, the yield is still around 50%. The systematic study of Copy Number Variants (CNVs) could contribute to improving our diagnostic capacity. These alterations have already been described as responsible for cardiomyopathies in some cases; however, their impact has been rarely assessed. We analyzed the clinical significance of CNVs in cardiomyopathies by studying 11,647 affected patients, many more than those considered in previously published studies. We evaluated the yield of the systematic study of CNVs in a production context using NGS and a novel CNV detection software tool v2.0 that has demonstrated great efficacy, maximizing sensitivity and avoiding false positives. We obtained a CNV analysis yield of 0.8% that fluctuated depending on the type of cardiomyopathy studied (0.29% HCM, 1.41% DCM, 1.88% ARVC, 1.8% LVNC, 1.45% RCM), and we present the frequency of occurrence for 18 genes that agglutinate the 95 pathogenic/likely pathogenic CNVs detected. We conclude the importance of including in diagnostic tests a systematic study of these genetic alterations for the different cardiomyopathies.

## 1. Introduction

Cardiomyopathies are disorders in which the heart muscle presents structural and functional abnormalities in the absence of underlying coronary artery disease, hypertension, valvular disease, and congenital disease [1]. These disorders are one of the main cardiac diseases and causes of sudden death among young people. They usually have genetic origin and familial presentation. This heterogeneous group of diseases has incomplete penetration and overlapping phenotypes [2,3,4,5].

In the last 30 years, there has been excellent progress made in the study and understanding of these diseases, from being considered idiopathic to establishing a classification based on the underlying molecular and genetic causes [2]. The European Society of Cardiology distinguishes five classes of cardiomyopathies: Hypertrophic Cardiomyopathy (HCM), Dilated Cardiomyopathy (DCM), Non-Dilated Left Ventricular Cardiomyopathy (NDLVC), Arrhythmogenic Right Ventricular Dysplasia (ARVC), Restrictive Cardiomyopathy (RCM), and finally a group of cardiomyopathies still to be classified that includes Non-Compaction Cardiomyopathy (LVNC) [2,3,6]. The prevalence of these diseases varies from one case in 250–500 persons for HCM to one case in 2000–5000 persons for ARVC [2,3,4].

With the advent of NGS and its standardization in genetic studies during the last decade, the number of point and indel genetic variants known to be the cause of the different cardiomyopathies has increased. The number of genes associated with and evaluated for each of these diseases has also increased; however, the yield of the genetic diagnosis of cardiomyopathies is still around 50% depending on the type of cardiomyopathy involved [5,7]. For many families, genetic testing still does not identify any pathogenic variant associated with the disease, which may imply that alternative genetic causes have not been evaluated, including the presence of CNVs and structural variants [7,8,9].

CNVs, which are DNA segments that have variable copy numbers with respect to the reference genome, are known to be one of the main sources of genetic variation in an individual [10,11]. Some of these CNVs are associated with diseases, including cardiomyopathies. In 1992, a CNV (a partial deletion) in *MYH7* was published as a possible cause of HCM development [12]. The number of characterized and clinically relevant CNVs in the development of cardiomyopathies and other diseases has not stopped growing in recent decades and has experienced a boost with NGS [9,13,14]. In 2020, our group published two cases of LVNC associated with 1p36 deletion syndrome [15]. Also, in 2020, the first three studies including families with CNVs in FHOD3 associated with HCM, a gene very recently associated with this disease, were published [7].

In an effort to approximate aggregate information on characterized CNVs, the DGV and DECIPHER databases were created, the latter focusing on disease-associated CNVs. On the other hand, the ACMGs updated its guidelines with special reference to the clinical evaluation of CNVs; however, despite the evident relevance of these genetic alterations, their systematic analysis in genetic testing is still not a reality. Determining the clinical relevance of these alterations is complex; moreover, only recently have some studies been carried out attempting to determine the expected yield of their study in the field of cardiomyopathies.

## 2. Materials and Methods

A cohort of 11,647 unrelated patients was studied. Racial origin was available for 84% of the patients, with 99% of them being Western Caucasian. Nationality was recorded in 95% of the cases, mainly Spanish (70%) and British (14%). The samples were referred from 360 hospitals from different parts of the world, including London and several Spanish autonomous communities as the main sources.

The study was conducted in accordance with the Declaration of Helsinki and approved by the Ethics Committee of A Coruña—Ferrol on 21 December 2020 (protocol code 2019/437). Informed consent was obtained from all subjects involved in the study.

**Figure 1:**
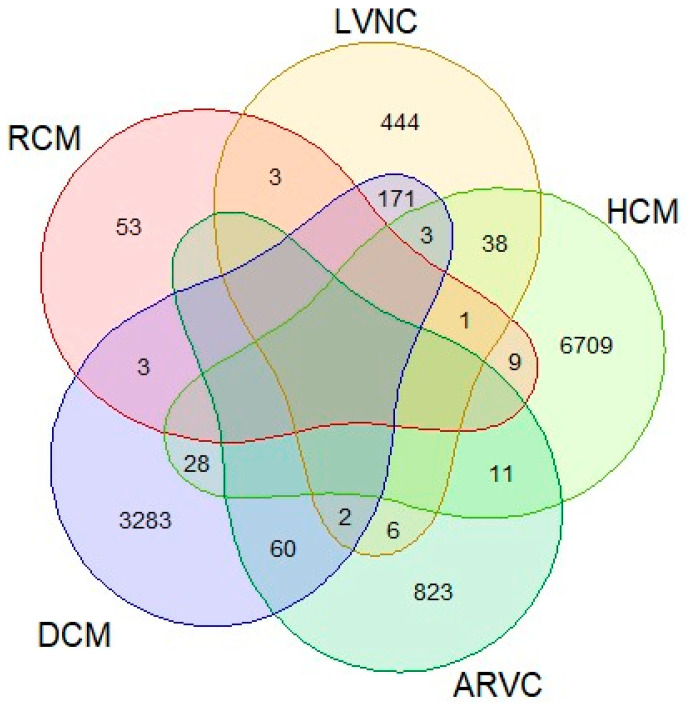
Phenotypes indicated by a clinician for patients as being affected or possibly affected.

All patients were referred by clinical specialists for study, indicating confirmed or probable involvement of cardiomyopathy: 6799 HCM, 3550 DCM, 902 ARVC, 668 LVNC, and 69 RCM. Sometimes, several overlapping phenotypes were indicated for the same patient (Figure 1).

For each patient included in the study, a panel of 170 genes related to hereditary heart disease was analyzed by means of NGS. The selection of genes was mainly based on the literature. We also considered the representation of each gene by phenotype in our database and the information available in the ClinGen resource [16]. For each gene, the coding and flanking regions at 10 bp were studied considering all the transcripts described in the RefSeq project database [17]. The list of genes studied is shown in Table 1.

For the sequencing of the samples, a personalized capture library was prepared according to the regions of interest described using Agilent’s SureSelect Target Enrichment technology. During the probe design process, the SureDesign tool from the same company was used. The samples were processed in the laboratory following the indications of the protocol described by the manufacturer for SureSelect. Paired-end sequencing was performed using the HiSeq 1500 platform from Illumina.

The average number of times each DNA base was captured and sequenced per study (coverage depth) was in the range [250–400×]. It was ensured that each base was sequenced at a depth equal to or greater than 30×; otherwise, the necessary regions (<0.5% of the bases) were additionally sequenced via the Sanger method.

Point variants/indels and CNVs were analyzed for each sample using algorithms optimized for the described experimental design. Sequences were aligned against the GRCh37 reference genome. Specific treatment was included for areas of high homology, as well as base recalibration and duplicate removal among other features. Point variants were detected using several genotypers and annotated with different databases prior to their study.

**Figure 2:**
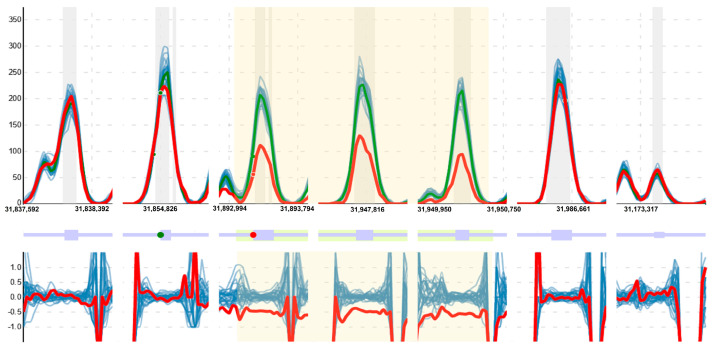
Example of the visualization provided by the CNV detection tool for one of the CNVs detected, in this case in the *DMD* gene. The software (v2.0) reported a deletion in heterozygosis for a female with very high confidence (region in yellow). The green line represents the normalized reading depth expected from a model generated with control samples (in blue); the case study is shown in red. The upper part of the graph shows the normalized reading depth, the middle part shows a plot of the gene located in the region, and the lower part shows the ratio of each sample to the model. Note that, in the part highlighted in yellow, there is a decrease in the signal that the program reports at 50%, the expected ratio for a deletion in heterozygosis. In addition, the point variants detected are presented as dots. Point variants detected in homozygosis are shown in red and heterozygous variants in green. In this example, the case has a variant in homozygosis in the selected region versus one in heterozygosis for one of the controls at the same position, also consistent with the reported CNV.

For the detection of CNVs, a method based on the comparison of the reading depth obtained for each base in the study sample against a series of control samples was used after a normalization and selection process [18]. The algorithm selects samples through a clustering process that considers the similarity of laboratory conditions and the fit of reading depth profiles. It then generates a reference model and detects regions where the normalized reading depth of the case differs significantly from that expected according to the model; these regions are extended and evaluated to determine whether the observed reading depth variations are related to the gains or losses of genetic material. During the evaluation, parameters such as the number of positive tests (the deviation is studied for each nucleotide base), the degree of deviation, the gain or loss ratio relative to the model, as well as its stability along the study region and other characteristics, such as the presence of breakpoints in the transition zones, are considered. As part of the results, all this information is displayed for each sample in a graphical and interactive way, allowing for the examination of all these features, as well as the presence of point variants and their zygosity for each region reported; see Figure 2.

All the detected point variants/indels and CNVs were classified, and their pathogenicity was evaluated by a clinical specialist following the guidelines established by the American College of Medical Genetics and Genomics (ACMGs) [19]. In addition, variants reported as relevant, including CNVs, and reported as associated with the patient’s phenotype, were confirmed via another technique complementary to NGS depending on their nature, such as SNP arrays, MLPA, digital PCR, or Sanger. Some of the CNVs detected covered several genes; in these cases, we used an SNP array to identify the genes affected by the event and took this information into account when evaluating the variant.

## 3. Results

For 3632 of the 11,647 patients studied, the result of the genetic study was positive; i.e., a pathogenic or likely pathogenic variant according to the ACMGs’ standards [19,20] was identified and explained the patient’s phenotype. The rest of the studies were negative or inconclusive. In 95 of the positive studies, the genetic variant associated with the disease was a CNV, accounting for 2.6% of the positive studies and 0.8% of the total number of studies performed. Details of each of the 95 CNVs (pathogenic or likely pathogenic) that were associated with the phenotype can be found in the Supplementary Materials, Table S1.

The number of CNVs associated with the disease by phenotype and their contribution to the total number of positive studies are listed below: 20 (0.9%) HCM, 50 (4.97%) DCM, 17 (5.74%) ARVC, 12 (5.8%) LVNC, and 1 (3.7%) RCM. More detailed data can be found in Table 2. Note that in terms of the total studies’ yields per pathology, the percentages explained by the CNVs detected were 0.82% considering all cardiomyopathies and, in particular, 0.29% HCM, 1.41% DCM, 1.88% ARVC, 1.8% LVNC, and 1.45% RCM.

Regarding the zygosity of the CNVs detected, 69 were heterozygous, 25 were hemizygous (all of them in males and in the *DMD* gene located on the X chromosome), and finally, there was one mosaicism with an alternative allele frequency observed close to 50% in a male and also in the *DMD* gene.

Some of the CNVs detected ranged from small regions, where we have only sequenced tens of nucleotides (for example, a 20bp exon deletion in *FLNC*), to several megabases (such as the deletion of the 2q11.21 region). Their typology was 72 deletions, 11 duplications (one of them of 4 copies), and 11 deletion–insertions (deletions where, in addition to the loss of the wildtype sequence, a new sequence that we identified is introduced). Additionally, one of the patients had a complex rearrangement involving both a duplication and a deletion event (counted as one CNV in this study).

CNVs detected were located in 18 of the 170 genes studied. Most of them (62%) were in *DMD* (34), *PKP2* (14), and *MYBPC3* (11). Table 3 shows in detail the genes involved, specifying their frequency for each of the pathologies studied.

**Figure 3:**
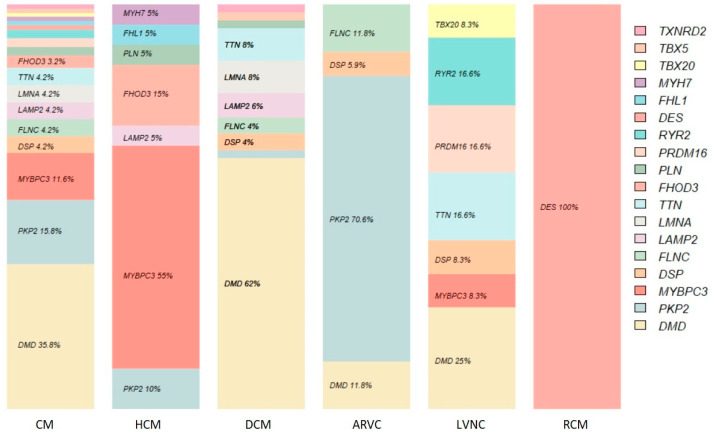
Relative frequency per gene with associated CNVs.

For HCM, 55% (11) of the associated CNVs involved the *MYBPC3* gene. For DCM, 62% (31) affected the *DMD* gene; in addition, 71% of CNVs in ARVC corresponded to *PKP2*. For LVNC, the CNVs (11) were not as concentrated in one gene, with 27% in *DMD* and 17% in *TTN*, *RYR2* (exon 3 deletion), and *PRDM16*. Finally, the CNV detected for RCM affected the *DES* gene. Figure 3 shows the percentages discussed extended to all CNVs and according to the frequency table.

The yields per gene were calculated from the data presented. For HCM, the highest yield was *MYBPC3* with 0.16% of the 6799 studies performed and 0.49% of the 2223 positive studies. For DCM, the CNVs in *DMD* explained 0.87% of the studies and 3.07% of the positive cases. Next, 1.33% of the ARVC studies were explained by CNVs in *PKP2* (4.05 of the positives). In LVNC, 0.45% of the studies presented a CNV in *DMD*, with 1.45% of the positives. In RCM, the case detected in *DES* accounted for 1.45% of the studies and 3.7% of the positive studies.

## 4. Discussion

The present study aims to gain a better understanding of the clinical value of incorporating CNV analysis in a systematic way in the study of hereditary cardiomyopathies.

Only a few studies have examined the contribution of CNVs in the study of these pathologies [8,9,14,21,22,23,24]; however, the size of the cohorts has not allowed more than 22 CNVs to be recorded in the largest study to date, which included 3233 patients [23]. For several of the pathologies, fewer than 100 patients were available. For this reason, all publications call for the continuation and expansion of such studies.

With 11,647 patients, with phenotypes characterized by a clinician for each of the pathologies studied and 95 pathogenic/likely pathogenic CNVs detected, the current study tripled the number of patients in the previous studies, allowing for a more comprehensive examination of each of the pathologies included.

The result of the genetic study was positive in 3632 patients belonging to our cohort, with a yield of about 30%, similar to that reported in the previous studies cited. We considered a study positive when we found a pathogenic or likely pathogenic variant that would explain the patient’s phenotype. The overall yield of the CNV analysis for all the cardiomyopathies was 0.8% for our cohort, close to the 0.7% described in the largest previous study, of which we are aware [23]; in other publications, it was estimated to be as high as 2.3 [22].

We observed a quite differentiated yield depending on the type of cardiomyopathy. For HCM, we obtained 0.29%, which is in the lower part of the [0.14–1.2] range suggested in previous studies [9,14,21,22,23]. In these studies, this pathology also presented a much lower yield than for DCM; for this phenotype, we obtained 1.41%, also within the range [0.6–2.2] described in the same previous studies.

As can be seen, there was a considerable difference between the yield values described in the previous studies, with the upper limit of the interval being almost ten times higher than the lower limit, for example, in HCM, no doubt due to the limited number of patients studied, which is consistent with the fact that the values calculated in this study are closer to those with larger cohorts.

For ARVC, LVNC, and RCM, the CNVs classified as pathogenic or likely pathogenic, explaining the patient’s phenotype, have been rarely reported in previous studies. For the RCM, we detected one CNV in 69 studies, a similar frequency result to that found in 52 patients described in a previous study [23]. For the ARVC, we obtained the highest yield for this pathology in the largest cohort so far (118 patients), which was 1.88% in 902 patients studied; here, five CNVs were reported as the cause of the disease, which was 4.2% [22]. Finally, we detected 12 pathogenic/likely pathogenic CNVs in the 668 patients for LVNC, whereas previous studies, with less than 150 patients with the phenotype, detected one or no pathogenic CNVs and thus returned yields of less than 1.65%, presumably due to a too small n-value and the introduction of new genes, such as *TBX20* and *PRDM16*.

The data presented in this study are consistent with previously available information, extend the knowledge for phenotypes with few studies, and refine the available information in terms of expected yields.

In addition to assessing the yield of CNV analysis for each type of cardiomyopathy, a map of the genes in which pathogenic CNVs were located is also presented. Having studied 170 genes, the fact that the CNVs were concentrated in 18 of them and mostly in about 10 genes provides new information, probably reflecting that there are specific genes intolerant to haploinsufficiency, and where loss-of-function is a definitive mechanism for the disease. Among the CNVs reported, a small percentage was duplications, mostly located in the DMD gene. These duplications were classified as likely pathogenic following the ACMGs’ guidelines and based on the evidence and the particular nature of the variant and the case. It is interesting to note that some duplications also lead to loss of function, for example, by altering the reading frame and causing premature stop codons. In particular, in *DMD*, there are clinical trials to test gene therapy based on exon skipping (e.g., *DMD* dup exon 2). So far, the panels studied were smaller, and the number of CNVs studied was less significant. We see that the pathogenic CNVs are mainly in the same genes that have already been described for the point variants and indels for each pathology.

Studies where no pathogenic or likely pathogenic variant was identified to explain the patient’s phenotype were considered negative in this work, even though they were originally reported as inconclusive because they found a Variant of Uncertain Significance (VUS). VUSs were not considered in this study, as the focus was on diagnosis, i.e., the number of positive results associating the cause of the disease with a CNV. However, it is interesting to note that during the study, we found 166 CNVs classified as VUSs (in addition to the 95 CNVs classified as pathogenic or likely pathogenic discussed in this study). As CNV analysis is not performed systematically, there is much less information available to assess CNVs; so, we believe it is important to routinely perform this type of analysis and generate new information that we believe could change the assessment of these variants and expand the current diagnostic capacity.

Although the overall yield of the CNV analysis was around 1%, this percentage was still higher than that obtained by adding new genes to a few panels. On the other hand, this study identified a limited priority set of genes after systematically screening 170 genes.

We found that for 2.6% of the positive cases, the genetic variant that explained the phenotype was a CNV. Ninety-five patients were able to benefit from the diagnosis due to the systematic CNV analysis. Although this type of analysis for NGS is less standardized than the approach for the detection of point variants and indels, a powerful system for CNV detection has been presented and proven to be effective. On the other hand, this analysis is performed on the same laboratory data used for the detection of point variants and indels.

We believe that the presented diagnostic power of CNV analysis and the available methods justify its systematic study in clinical practice for cardiomyopathies.

## Figures and Tables

**Table 1:** List of genes included in the study, taking the coding regions with 10 bp flanking for the transcripts described in the NCBI RefSeq project.

Genes Studied						
*AARS2*	*ABCC9*	*ACAD9*	*ACADVL*	*ACTA1*	*ACTC1*	*ACTN2*
AGK	*AGL*	*AGPAT2*	*AKAP9*	*ALMS1*	*ANK2*	*ANK3*
*ANKRD1*	*ATPAF2*	*BAG3*	*BRAF*	*BSCL2*	*CACNA1C*	*CACNA1D*
*CACNA2D1*	*CACNB2*	*CALM1*	*CALM2*	*CALR3*	*CAPN3*	*CASQ2*
*CAV3*	*CAVIN4*	*COQ2*	*COX15*	*COX6B1*	*CRYAB*	*CSRP3*
*CTNNA3*	*DES*	*DLD*	*DMD*	*DNAJC19*	*DOLK*	*DSC2*
*DSG2*	*DSP*	*DTNA*	*EMD*	*EYA4*	*FAH*	*FHL1*
*FHL2*	*FHOD3*	*FKRP*	*FKTN*	*FLNC*	*FOXD4*	*GAA*
*GATA4*	*GATA6*	*GATAD1*	*GFM1*	*GJA1*	*GJA5*	*GLA*
*GLB1*	*GNPTAB*	*GPD1L*	*GUSB*	*HCN4*	*HFE*	*HRAS*
*JPH2*	*JUP*	*KCNA5*	*KCND3*	*KCNE1*	*KCNE2*	*KCNE3*
*KCNE5*	*KCNH2*	*KCNJ2*	*KCNJ5*	*KCNJ8*	*KCNK3*	*KCNQ1*
*KLF10*	*KRAS*	*LAMA2*	*LAMA4*	*LAMP2*	*LDB3*	*LDLR*
*LIAS*	*LMNA*	*MAP2K1*	*MAP2K2*	*MIB1*	*MLYCD*	*MRPL3*
*MRPS22*	*MTO1*	*MYBPC3*	*MYH11*	*MYH6*	*MYH7*	*MYL2*
*MYL3*	*MYLK2*	*MYOT*	*MYOZ2*	*MYPN*	*NEBL*	*NEXN*
*NKX2-5*	*NOTCH1*	*NPPA*	*NRAS*	*OBSL1*	*PDHA1*	*PDLIM3*
*PHKA1*	*PITX2*	*PKP2*	*PLN*	*PMM2*	*PRDM16*	*PRKAG2*
*PSEN1*	*PSEN2*	*PTPN11*	*RAF1*	*RANGRF*	*RBM20*	*RYR2*
*SCN10A*	*SCN1B*	*SCN2B*	*SCN3B*	*SCN4B*	*SCN5A*	*SGCA*
*SGCB*	*SGCD*	*SHOC2*	*SLC22A5*	*SLC25A4*	*SLMAP*	*SNTA1*
*SOS1*	*SPRED1*	*SRY*	*SURF1*	*TAZ*	*TBX20*	*TBX5*
*TCAP*	*TGFB3*	*TMEM43*	*TMEM70*	*TNNC1*	*TNNI3*	*TNNT2*
*TPM1*	*TRDN*	*TRIM63*	*TRPM4*	*TSFM*	*TTN*	*TTR*
*TXNRD2*	*VCL*					

**Table 2:** Number of patients studied by pathology and in total for cardiomyopathies (CMs). Distribution according to whether the study was negative or positive. In the case of positive studies, it is indicated in how many of them the variant related to the disease was a CNV. For the purposes of this classification, we also consider inconclusive genetic studies as negative. The percentage of studies where the pathogenic or likely pathogenic variant is a CNV is calculated for the total and with respect to the positive studies.

Study	CNVs	CM	HCM	DCM	ARVC	LVNC	RCM
+	+	95	20	50	17	12	1
+	−	3537	2203	957	279	195	26
−	−	8015	4576	2543	606	461	42
Patients	studied	11,647	6799	2550	902	668	69
%CNVs+	(total)	0.82	0.29	1.41	1.88	1.80	1.45
%CNVs+	(study+)	2.62	0.90	4.97	5.74	5.80	3.70

**Table 3:** Detail of the genes involved in the pathogenic/likely pathogenic CNVs studied, for the total of cardiomyopathies (CMs) and for each of the disorders. *TXNRD2* is placed as a representative gene within the panel for the CNV detected, which is much more extensive (chromosomal deletion 2q11.21). The CNVs in the *FHOD3* gene have been published [7]. Note that there are several CNVs that are included in the count for several pathologies, since the carrier patient has several associated phenotypes. Counts of 0 are left blank.

Gene	CM	HCM	DCM	ARVC	LVNC	RCM
*DMD*	34		31	2	3	
*PKP2*	14	2	1	12		
*MYBPC3*	11	11			1	
*DSP*	4		2	1	1	
*FLNC*	4		2	2		
*LAMP2*	4	1	3			
*LMNA*	4		4			
*TTN*	4		4		2	
*FHOD3*	3	3				
*PLN*	2	1	1			
*PRDM16*	2				2	
*RYR2*	2				2	
*DES*	1					1
*FHL1*	1	1				
*MYH7*	1	1				
*TNX20*	1				1	
*TBX5*	1		1			
*TXNRD2*	1		1			

## Data Availability

The raw data supporting the conclusions of this article will be made available by the authors on request.

## Supplementary Materials

The following supporting information can be downloaded at: https://www.mdpi.com/article/10.3390/genes15060774/s1, Table S1: List of the 95 CNVs.
