# Supplementary material for: Clinical Relevance of the Systematic Analysis of Copy Number Variants in the Genetic Study of Cardiomyopathies
Source: Genes (Basel). 2024 Jun 13;15(6):774. doi: 10.3390/genes15060774 (PMC11203228; doi:10.3390/genes15060774)
Supplement: Supplementary file 1 [file genes-15-00774-s001.zip › genes-3035211-supplementary.pdf]

GCAT TACG GCAT genes

MDPI

Supplementary Materials

Clinical relevance of the systematic analysis of CNVs in the genetic study of cardiomyopathies.

David de Uña-Iglesias [1,*], Juan Pablo Ochoa [2] , Lorenzo Monserrat [3] and Roberto Barriales-Villa [4]

[1] Universidad de A Coruña, Spain; Biotechnology Department, Health in Code; daviddeuna@gmail.com
[2] Instituto de Investigación Biomédica de A Coruña (INIBIC), Cardiology Department, Health in Code; juanpablo.ochoa@healthincode.com
[3] Medical Department Dilema Solutions, A Coruña, Spain; lorenzo.monserrat@naeviamedical.com
[4] Unidad de Cardiopatías Familiares, Instituto de Investigación Biomédica de A Coruña (INIBIC), Complexo Hospitalario de A Coruña, Servizo de Saúde (SERGAS), Universidade de A Coruña. A Coruña, Spain, Centro de Investigación Biomédica en Red (CIBERCV), Madrid, Spain; rbarrialesv@gmail.com

* Correspondence: daviddeuna@gmail.com

Table S1. List of the 95 CNVs pathogenic (P) or likely pathogenic (LP) according to the ACMG guidelines considered positive in the study.

Phenotype	Gene	Pathogenicity	Exons involved	zygosity	HGVS Names
DCM	*DMD*	P	48-51	hemi	NC_000023.10:g.31792079_31893492del<br>NM_004006.2:c.6913_7542del<br>NP_003997.1:p.Val2305_Lys2514del
DCM	*DMD*	LP	48-49	hemi	NC_000023.10:g.31849704_31911151delinsGGAATAGTAG-GAATAGTAGA<br>NM_004006.2:c.6913-17661_7200+5131delinsTCTACTA-TTCCTACTATTCC<br>NP_003997.1:p.Val2305_Lys2400del
DCM	*DMD*	P	45-48	hemi	NC_000023.10:g.31856965_32119775del<br>NM_004006.2:c.6438+115262_7099-2025del<br>NP_003997.1:p.Glu2147_Lys2366de
DCM	*DMD*	LP	2	hetero	NC_000023.10:g.33038257_33038318dup<br>NM_004006.2:c.32_93dup<br>NP_003997.1:p.Phe32Metfs*15
DCM	*DMD*	P	47-48	hemi	NC_000023.10:g.31893307_31947864del<br>NM_004006.2:c.6763_7098del<br>NP_003997.1:p.Leu2255_Lys2366del
DCM	*DMD*	P	3-9	hemi	NC_000023.10:g.32685760_32988401del<br>NM_004006.2:c.93+49857_961-22489del<br>NP_003997.1:p.Phe32_Gln320del
DCM	*DMD*	P	48-51	hetero	NC_000023.10:g.31792079_31893492del<br>NM_004006.2:c.6913_7542del<br>NP_003997.1:p.Val2305_Lys2514del
DCM	*DMD*	LP	19-43	hetero	NC_000023.10:g.32305646_32519959dup<br>NM_004006.2:c.2293_6290dup<br>NP_003997.1:p.?
DCM	*DMD*	P	49-51	hetero	NC_000023.10:g.31792079_31854938del<br>NM_004006.2:c.7099_7542del<br>NP_003997.1:p.Glu2367_Lys2514del

DCM	*DMD*	P	45	hetero	NC_000023.10:g.31986458_31986633del NM_004006.2:c.6439_6614del NP_003997.1:p.Glu2147Alafs*17
DCM	*DMD*	P	45-48	hemi	NC_000023.10:g.31893307_31986633del NM_004006.2:c.6439_7098del NP_003997.1:p.Glu2147_Lys2366del
DCM	*DMD*	LP	44-53	hetero	NC_000023.10:g.31697703_32235033del NM_004006.2:c.6438_7661del NP_003997.1:p.Lys2146_Ile2554delinsAsn
DCM	*DMD*	LP	56-59	hemi	NC_000023.10:g.31496223_31525570dup NM_004006.2:c.8218_8937dup
DCM	*DMD*	P	45-53	hetero	NC_000023.10:g.31641792_31947605del NM_004006.2:c.6912+112_8217+4002del NP_003997.1:p.Val2305_Gln2739del
DCM	*DMD*	P	20-24	hetero	NC_000006.11:g.7577967_7584099del NM_004415.3:c.2878-45_6604del NP_004406.2:p.Asp960Glnfs*16
DCM	*DMD*	P	46-47	hetero	NC_000023.10:g.31947715_31950346del NM_004006.2:c.6615_6912del NP_003997.1:p.Leu2206Phefs*16
DCM	*DMD*	P	45-55	hemi	NC_000023.10:g.31645790_31986631del NM_004006.2:c.6439_8217del NP_003997.1:p.Glu2147_Gln2739del
DCM	*DMD*	P	48-49	hemi	NC_000023.10:g.31845995_31904473del NM_004006.2:c.6913-10983_7201-7795del NP_003997.1:p.Val2305_Lys2400del
DCM	*DMD*	LP	48-51	hemi	NC_000023.10:g.31787841_31896426delinsCCTTTGAAC NM_004006.2:c.6913-2936_7542+4236delinsGTTCAAAGG NP_003997.1:p.?
DCM	*DMD*	LP	19-25	hemi	NC_000023.10:g.32474947_32524395dup NM_004006.2:c.2293-4436_3433-1998dup NP_003997.1:p.?
DCM	*DMD*	P	48-49	hemi	NC_000023.10:g.31854837_31893492del NM_004006.2:c.6913_7200del NP_003997.1:p.Val2305_Lys2400del
DCM	*DMD*	LP	35-44	hemi	NC_000023.10:g.32177150_32390103del NM_004006.2:c.4846-6787_6438+57883del NP_003997.1:p.Ala1616_Lys2146del
DCM	*DMD*	P	46-48	hetero	NC_000023.10:g.31869704_31954286del NM_004006.2:c.6615-3939_7099-14765del NP_003997.1:p.Leu2206Lysfs*4
DCM	*DMD*	P	52-53	hemi	NC_000023.10:g.31697494_31747867del NM_004006.2:c.7543_7872del NP_003997.1:p.Ala2515_Lys2624de
DCM	*DMD*	P	48-49	hemi	NC_000023.10:g.31854837_31893492del NM_004006.2:c.6913_7200del NP_003997.1:p.Val2305_Lys2400de

DCM	*DMD*	P	45-48	hemi	NC_000023.10:g.31893307_31986633del<br>NM_004006.2:c.6439_7098del<br>NP_003997.1:p.Glu2147_Lys2366del
DCM	*DMD*	P	48-51	hemi	NC_000023.10:g.31792079_31893492del<br>NM_004006.2:c.6913_7542del<br>NP_003997.1:p.Val2305_Lys2514del
DCM	*DMD*	P	45-55	hemi	NC_000023.10:g.31645790_31986631del<br>NM_004006.2:c.6439_8217del<br>NP_003997.1:p.Glu2147_Gln2739del
DCM	*DMD*	P	45-53	hemi	NC_000023.10:g.31697494_31986633del<br>NM_004006.2:c.6439_7872del<br>NP_003997.1:p.Glu2147_Lys2624del
DCM	*DMD*	LP	67-74	hetero	NC_000023.10:g.31175526_31235032delins<br>[NC_000011.9:g.2434 9496-24355552]<br>NM_004006.2:c.9361+6132_10554-9891delins<br>NP_003997.1:p.Leu3121Serfs*11
DCM	*DSP*	LP	1-2	hetero	NC_000006.11:g.7542149_7556053del<br>NM_004415.3:c.1_273del<br>NP_004406.2:p.0?
DCM	*FLNC*	LP	3-5	hetero	NC_000007.13:g.128476527_128477737del<br>NM_001458.4:c.602-687_897del<br>NP_001449.3:p.?
DCM	*FLNC*	LP	24-32	hetero	NC_000007.13:g.128486595_128490469del<br>NM_001458.4:c.4127+77_5329del<br>NP_001449.3:p.?
DCM	*LAMP2*	LP	all	hetero	NC_000023.10:g.119562339_119590624del<br>NM_002294.2:c.65_*2839del<br>NP_002285.1:p.?
DCM	*LAMP2*	LP	6-8	hetero	NC_000023.10:g.119575587_119580284del<br>NM_002294.2:c.742_1093del<br>NP_002285.1:p.Val248Leufs*15
DCM	*LAMP2*	LP	1-8	hetero	NC_000023.10:g.119573044_119636423delins174<br>NM_002294.2:c.-33399_1093+2541delins174<br>NP_002285.1:p.Met1Glyfs*11
DCM	*LMNA*	P	2	hetero	NC_000001.10:g.156099922_156102230del<br>NM_170707.3:c.357-487_513+1665del<br>NP_733821.1:p.Asn120Leufs*5
DCM	*LMNA*	LP	all	hetero	NC_000001.10:g.156052337_156109880del<br>NM_170707.3:c.-32373_*983del<br>NP_733821.1:p.0?
DCM	*LMNA*	P	2-12	hetero	NC_000001.10:g.156095595_156111755del<br>NM_170707.3:c.357-4813_*2858del<br>NP_733821.1:p.Arg119fs
DCM	*LMNA*	LP	5-10	hetero	NC_000001.10:156104900_156108240dup<br>NM_170707.3:c.811-78_1699-39dup
DCM	*PKP2*	LP	all	hetero	NC_000012.11:g.32943680_33049780del<br>NM_004572.3:c.-115_*1678del<br>NP_004563.2:p.Met1_*882del

DCM	*PLN*	LP	all	hetero	NC_000006.11:g.(?_118869427)_(118880244_?)x4
DCM	*TBX5*	LP	6	hetero	NC_000012.11:g.114832548_114832700del<br>NM_000192.3:c.511_663del<br>NP_000183.2:p.Ile171_Lys221del
DCM	*TTN*	LP	154	hetero	NC_000002.11:g.179433230_179436528delinsTGCGA-TAACATTAAGGGTTTCAATAGCTTCACCAGCTGAGTTAGTCA-GTTTAACCACATAATGGCCAACATCTTCTCGGCAGGC<br>NM_003319.4:c.47136_50434delinsGCCTGCCGAGAA-GATGTTGGCCATTATGTGGTTAAACTGACTAACTCA-GCTGGTGAAGCTATTGAAACCCTTAATGTTATCGCA<br>NP_003310.4:p.Asp15712Glufs*14
DCM	*TTN*	LP	188-191<br>154-191	hetero	NC_000002.11:g.179391078_179393287dup<br>NM_003319.4:c.79996_*661dup<br>NP_003310.4:p.P26666_*26927dup.<br>NC_000002.11:g.179391452_179424996del<br>NM_003319.4:c.58668_*287del<br>NP_003310.4:p.Tyr19556fs
DCM	*TXNRD2* **2*	P	all*	hetero	NC_000022.10:g.19167732_21351637del<br>22q11.2del
DCM/LVNC	*TTN*	LP	154	hetero	NC_000002.11:g.179433230_179436528delins-GCCTGCCGAGAAGATGTTGGCCATTATGTGGTTAAACTGAC-TAACTCAGCTGGTGAAGCTATTGAAACCCTTAATGTTATCGCA<br>NM_003319.4:c.47136_50434delinsTGCGATAACAT-TAAGGGTTTCAATAGCTTCACCAGCTGAGTTAGTCAGTTTAAC-CACATAATGGCCAACATCTTCTCGGCAGGC<br>NP_003310.4:p.Cys15714Ilefs*19
DCM/LVNC	*TTN*	LP	154	hetero	NC_000002.11:g.179433230_179436528delins-GCCTGCCGAGAAGATGTTGGCCATTATGTGGTTAAACTGAC-TAACTCAGCTGGTGAAGCTATTGAAACCCTTAATGTTATCGCA<br>NM_003319.4:c.47136_50434delinsTGCGATAACAT-TAAGGGTTTCAATAGCTTCACCAGCTGAGTTAGTCAGTTTAAC-CACATAATGGCCAACATCTTCTCGGCAGGC<br>NP_003310.4:p.Cys15714Ilefs*19
DCM/LVNC	*DMD*	LP	48-51	hemi	NC_000023.10:g.31792079_31893492de<br>NM_004006.2:c.6913_7542del<br>NP_003997.1:p.Val2305_Lys2514del
DCM/LVNC	*DMD*	LP	26-42	hemi	NC_000023.10:g.32328203_32472953del<br>NM_004006.2:c.3433_6117del<br>NP_003997.1:p.Val1145_Lys2039del
LVNC	*DMD*	P	49	hetero	NC_000023.10:g.31845186_31878210del<br>NM_004006.2:c.7098+15096_7201-6985del<br>NP_003997.1:p.Glu2367_Lys2400del
LVNC	*DSP*	LP	12	hetero	NC_000006.11:g. 7569421_7569481dup<br>NM_004415.3:c.1422_1482dup<br>NP_004406.2:p.Val495Asnfs*4
LVNC	*PRDM16* **1*	P	all	hetero	1p36.33-p36.32 del (4Mb)

LVNC	*PRDM16*	LP	2-17	hetero	NC_000001.10:g.3102689_3350375del NM_022114.3:c.38_3831del NP_071397.3:p.*12fs
LVNC	*RYR2*	LP	3	hetero	NC_000001.10:g.237494234_237503798del NM_001035.2:c.223_273+9514del NP_001026.2:p.Val75_Gly91del
LVNC	*RYR2*	LP	4	hetero	NC_000001.10:g.237519265_237519285del NM_001035.2:c.274_294del NP_001026.2:p.Gln92_Trp98del
LVNC	*TBX20*	LP	7-8	hetero	NC_000007.13:g.35242042_35244194del NM_001077653.2:c.891_1344del NP_001071121.1:p.Arg297(?)
LVNC/HCM	*MYBPC3*	LP	21	hetero	NC_000011.9:g.47361014_47362361del NM_000256.3:c.1927+193_2068-59del NP_000247.2:p.Glu643Glyfs*3
HCM	*FHL1*	LP	all	hetero	NC_000023.10:g.135278961_135292184del NM_001159700.1:c.-26-9605_843del NP_001153172.1:p.0?
HCM	*FHOD3*	P	15	hetero	NC_000018.9:g.34260087_34262764delinsGTTCTGCCCA-TAGTCTTTGCTCCCAGG NM_001281740.2:c.1836-1312_1970+1231delinsTTCTGCC-CATAGTCTTTGCTCCCAGG NP_001268669.1:p.Arg612_Phe656del
HCM	*FHOD3*	P	15-16	hetero	NC_000018.9:g.34259872_34271130del NM_001281740.2:c.1836-1527_2022-2042del NP_001268669.1: p.Ser613_Arg674del
HCM	*FHOD3*	P	15-16	hetero	NC_000018.9:g.34258856_34269358delinsT-GTAAAAAAAAAATAAAAGATATAAAGATCCAAATGGAAATA-TATATAGAACAG-GAAAATTAAAATAGAAAATTTGTCAGATGGGCCAAATAGAATTGCTATGA NM_001281740.2:c.1836-2543_2021+2217delinsTGTAAAAAAAAAATAAAAGATA-TAAAGATCCAAATGGAAATATATATAGAACAG-GAAAATTAAAATAGAAAATTTGTCAGATGGGCCAAATAGAATTGCTATGAG NP_001268669.1:p.Ser613_Arg674del
HCM	*LAMP2*	LP	7	hetero	NC_000023.10:g.119576455_119576518del NM_002294.2:c.865_928del NP_002285.1:p.Lys289Phefs*36
HCM	*MYBPC3*	LP	4-5	hetero	NC_000011.9:g.47371423_47372009del NM_000256.3:c.406+45_557del NP_000247.2:p.Ser137Leufs*13
HCM	*MYBPC3*	LP	1-5	hetero	NC_000011.9:g.47370237_47375203del NM_000256.3:c.-999_655-139del NP_000247.2:p.0?
HCM	*MYBPC3*	LP	4-7	hetero	NC_000011.9:g.47369431_47371766del NM_000256.3:c.407-103_798del NP_000247.2:p.?

HCM	*MYBPC3* *3	LP	all	hetero	NC_000011.9:g.34196603_49219155del
HCM	*MYBPC3*	LP	1-17	hetero	NC_000011.9:g.47364046_47375171del<br>NM_000256.3:c.-973_1624+83del<br>NP_000247.2:p.0?
HCM	*MYBPC3*	LP	18	hetero	NC_000011.9:g.47363206_47363745del<br>NM_000256.3:c.1625-38_1790+336del<br>NP_000247.2:p.Glu542Glyfs*5
HCM	*MYBPC3*	P	23-26	hetero	NC_000011.9:g.47357175_47360721del<br>NM_000256.3:c.2148+154_2737+253del<br>NP_000247.2:p.Leu717Alafs*11
HCM	*MYBPC3*	P	23-26	hetero	NC_000011.9:g.47357175_47360721del<br>NM_000256.3:c.2148+154_2737+253del<br>NP_000247.2:p.Leu717Alafs*11
HCM	*MYBPC3*	LP	27-7	hetero	NC_000011.9:g.47356553_47357279delinsC<br>NM_000256.3:c.2737+149_2905+40delinsG<br>NP_000247.2:p?
HCM	*MYBPC3*	LP	27	hetero	NC_000011.9:g.47356553_47357279delinsC<br>NM_000256.3:c.2737+149_2905+40delinsG<br>NP_000247.2:p.?
HCM	*MYH7*<br>*MYH6*	P	*MYH7* all<br>*MYH6* 1-5	hetero	NC_000014.8:g.23874432_23901847del
HCM	*PKP2*	LP	13-14	hetero	NC_000012.11:g.32945358_32945665del<br>NM_004572.3:c.2490_2646del NP_004563.2:p.Ala830fs
HCM	*PKP2*	P	8	hetero	NC_000012.11:g.32976981_32977098del<br>NM_004572.3:c.1689_1806del<br>NP_004563.2:p.Asn564Profs*53
HCM	*PLN*	LP	all	hetero	NC_000006.11:g.118869442_118880243del<br>NM_002667.3:c.-211_159del<br>NP_002658.1:p.0?
RCM	*DES*	LP	1-4	hetero	NC_000002.11:g.220267400_220285223dupinv<br>NM_001927.3:c.-15785_742dupinv
ARVC	*DMD*	LP	25-30	hemi	NC_000023.10:g.32423738_32481666del<br>NM_004006.2:c.3322_4233+6131del<br>NP_003997.1:p.Val1108_Gln1411del
ARVC	*DMD*	LP	40-55	hemi	NC_000023.10:g.31645790_32361403dup<br>NM_004006.2:c.5587_8217dup<br>NP_003997.1:p.?
ARVC	*DSP*	P	21-24	hetero	NC_000006.11:g.7577966_7584094del<br>NM_004415.2:c.2878-46_6599del<br>NP_004406.2:p.Asp960Alafs*21
ARVC	*FLNC*	LP	all	hetero	NC_000007.13:g.128470694_128498579del<br>NM_001458.4:c.3_*2del<br>NP_001449.3:p.0?
ARVC	*FLNC*	P	FLNC 3-48	hetero	NC_000007.13:g.128476955_128510390del<br>NM_001458.4:c.602-259_*11813del<br>NP_001449.3:p.?

ARVC	*PKP2*	LP	8	hetero	NC_000012.11:g.32976092_32978401del NM_004572.3:c.1689-1305_1807-527del NP_004563.2:p.Asn564Profs*53
ARVC	*PKP2*	LP	4	hetero	NC_000012.11:g.33019241_33026724del NM_004572.3:c.1034+4057_1170+2621del NP_004563.2:p.Asn346Leufs*14
ARVC	*PKP2*	LP	8	hetero	NC_000012.11:g.32976092_32978401del NM_004572.3:c.1689-1305_1807-527del NP_004563.2:p.Asn564Profs*53
ARVC	*PKP2*	P	8	hetero	NC_000012.11:g.32976092_32978401del NM_004572.3:c.1689-1305_1807-527del NP_004563.2:p.Asn564Profs*53
ARVC	*PKP2*	P	8	hetero	NC_000012.11:g.32976092_32978401del NM_004572.3:c.1689-1305_1807-527del NP_004563.2:p.Asn564Profs*53
ARVC	*PKP2*	LP	10	hetero	NC_000012.11:g.32974274_32974845del NM_004572.3:c.1972-380_2145+18del NP_004563.2:p.Gln658_Pro715del
ARVC	*PKP2*	LP	8-10	hetero	NC_000012.11:g.32974290_32977096dup NM_004572.3:c.1689_2145dup
ARVC	*PKP2*	LP	5-14	hetero	NC_000012.11:g.32924017_33011214delinsCATTAT NM_004572.3:c.1171-7307_*21341delinsATAATG NP_004563.2:p.?
ARVC	*PKP2*	LP	13-14	hetero	NC_000012.11:g.32942317_32945702del NM_004572.3:c.2490-35_*3043del NP_004563.2:p.Ala830fs
ARVC	*PKP2*	LP	13-14	hetero	NC_000012.11:g.32945358_32945665del NM_004572.3:c.2490_2646del NP_004563.2:p.Ala830fs
ARVC	*PKP2*	LP	8-10	hetero	NC_000012.11:g.32974290_32977096dup NM_004572.3:c.1689_2145dup
ARVC	*PKP2*	P	8	hetero	NC_000012.11:g.32976092_32978401del NM_004572.3:c.1689-1305_1807-527del NP_004563.2:p.Asn564Profs*53

*1 This deletion affects 112 genes (79 coding). The following are particularly relevant: *GNB1, ISG15, AGRN, TNFRSF4, B3GALT6 DVL1, ATAD3A, TMEM240, GABRD SKI, PEX10, PRDM16, SMIM1* and *CEP104*

*2 This deletions affects 13 genes: *CLTCL1, HIRA, CDC45, CLDN5, GP1BB, TBX1, TNXRD2, DGCR8, ZNF74, KLHL22, MED15, SNAP29* and *LZTR1*

*3 This deletion affects 158 genes (*MYBPC3* included), 85 are coding: *ABTB2, ACCS, ACCSL, ACP2, AGBL2, ALKBH3, ALX4, AMBRA1, API5, APIP, ARFGAP2, ARHGAP1, ATG13, C11orf49, C11orf74, C11orf94, C11orf96, C1QTNF4, CAT, CD44, CD82, CELF1, CHRM4, CHST1, CKAP5, COMMD9, CREB3L1, CRY2, DDB2, DGKZ, EHF, ELF5, EXT2, F2, FAM180B, FJX1, FNBP4, FOLH1, HARBI1, HSD17B12, KBTBD4, LARGE2, LDLRAD3, LRP4, LRRC4C, MADD, MAPK8IP1, MDK, MTCH2, MYBPC3, NDUFS3, NR1H3, NUP160, OR4A47, OR4B1, OR4C3, OR4S1, OR4X1, OR4X2, PACSIN3, PAMR1, PDHX, PEX16, PHF21A, PRDM11, PRR5L, PSMC3, PTPMT1, PTPRJ, RAG1, RAG2, RAPSN, SLC1A2, SLC35C1, SLC39A13, SPI1, SYT13, TP53I11, TRAF6, TRIM44, TRIM49B, TRIM64C, TSPAN18, TTC17, ZNF408*
